# Key Aspects of Amadori Rearrangement Products as Future Food Additives †

**DOI:** 10.3390/molecules26144314

**Published:** 2021-07-16

**Authors:** Yue Luo, Shiming Li, Chi-Tang Ho

**Affiliations:** 1 Department of Food Science, Rutgers University, New Brunswick, NJ 08901, USA; yue.luo@rutgers.edu (Y.L.); Shiming@rutgers.edu (S.L.); 2 Hubei Key Laboratory for EFGIR, Huanggang Normal University, Huanggang 438000, China

**Keywords:** flavor, Amadori rearrangement products, flavor additives, taste enhancing

## Abstract

Flavor is one of the most important factors in attracting consumers and maximizing food quality, and the Maillard reaction (MR) is highly-involved in flavor formation. However, Maillard reaction products have a big drawback in their relatively low stability in thermal treatment and storage. Amadori rearrangement products (ARPs), MR intermediates, can alternatively act as potential flavor additives for their better stability and fresh flavor formation ability. This review aims to elucidate key aspects of ARPs’ future application as flavorings. The development of current analytical technologies enables the precise characterization of ARPs, while advanced preparation methods such as synthesis, separation and drying processes can increase the yield of ARPs to up to 95%. The stability of ARPs is influenced by their chemical nature, pH value, temperature, water activity and food matrix. ARPs are associated with umami and kokumi taste enhancing effects, and the flavor formation is related to amino acids/peptides of the ARPs. Peptide-ARPs can generate peptide-specific flavors, such as: 1,6-dimethy-2(1H)-pyrazinone, 1,5-dimethy-2(1H)-pyrazinone, and 1,5,6-trimethy-2(1H)-pyrazinone. However, further research on systematic stability and toxicology are needed.

## 1. Introduction

The Maillard reaction has been one of the most important reactions in flavor generation since its first discovery by French chemist Louis-Camille Maillard in 1912 [1]. The sophisticated reaction cascades start with the formation of a Schiff base (glycosylamine) from the condensation reaction between the carbonyl groups of reducing sugars and amino groups of amino acids, peptides or proteins. The Schiff base then goes through Amadori rearrangement with a nucleophilic catalyst and forms a more stable 1-amino-1-deoxy-2-ketose, called Amadori rearrangement products (ARPs) [2]. While ARPs are specific for a Schiff base derived from aldose sugar, Heyns rearrangement products (HRPs, 2-amino-2-deoxyaldose) are derived from ketose sugar [3]. The successive breakdown of ARPs or HRPs generates abundant volatile carbocyclic and heterocyclic compounds, oligomers and polymers, thus bring various flavor and yellowish to brown colors to food products [4].

The Maillard reaction is commonly seen in thermally processed food with attractive and complex flavors, such as bread, cereal, coffee, roasted meat. These flavors mainly come from the formation of pyrazines, pyrroles, alkylpyridines, acylpyridines, furanones, furans, oxazoles, and thiophenes [5]. Each gives a unique flavor of cooked food. However, these Maillard reaction-derived flavors are of unsatisfactory stability. Food products likely go through high temperature processing like boiling, baking, frying or pasteurization and then a specific shelf life range from a few days to years before consumption. The loss of attractive flavor compounds such as acetaldehyde, furfural and butanal, has been a major concern regarding flavor quality and consumer acceptance [6]. Enormous efforts have been reported to stabilize flavor compounds, and among which nanoencapsulation is considered an efficient method [7]. Not until recently, have people started to pay attention to flavorless intermediates, ARPs. A big advantage of ARPs is better stability during storage and synchronous production of fresh and desirable flavors during thermal treatment, which makes them potential flavor enhancers and food additives. However, to our best knowledge, there is no comprehensive review on ARPs as potential food additives. Therefore, this review aims to, for the first time, elucidate aspects related to ARPs and discuss the possibility of being food additives.

For convenience, the ARP of amino acid/peptide and reducing sugar starting now is referred to as amino acid/peptide-reducing sugar-ARP. The ARP would share a similar chemical structure, named as *N*-(1-deoxy-d-fructos-1-yl)-amino acid/peptide (amino acid-glucose system) after the Amadori rearrangement. Some papers adopt abbreviations like fru-val for *N*-(1-deoxy-d-fructos-1-yl)-valine (herein after referred to as valine-glucose-ARP).

## 2. Analytical Methods

In early times, the analysis of the Maillard reaction was approximate and targeted at overall information of bulk change, using traditional analytical methods like fluorescence or absorbance measurement, furosine assay and fluorescamine assay among others [8,9,10]. Later, an amino acid analyzer was utilized, especially for the crystallized ARPs, with a satisfying purity [11]. The application of ninhydrin coupled with thin layer chromatography (TLC) turned out to be a good choice for preliminary screening. Though capable of analyzing ARPs by combination with the secondary amines, ninhydrin cannot exclude other compounds, such as amino acids [12,13].

As an effective analytical method, high-performance liquid chromatography (HPLC) or anion-exchange chromatography coupled with a diode array detector (DAD) and an evaporative light scattering detector (ELSD) is also largely used in the identification of ARPs [14,15]. However, most of these methods failed to achieve the unambiguous identification of each ARP with good peak shape and resolution. A primary reason is ascribed to the high polarity of ARPs, which cause early elution with numerous compounds in the reversed phase (RP) column and strong retention in the normal phase (NP) column. The recent development of the hydrophilic interaction liquid chromatography (HILIC) column acts as a good balance of RP and NP columns and enables a satisfying separation of polar compounds. Its application simultaneously separates glycine, diglycine, triglycine and their corresponding ARPs [16] and peptide ARPs [17].

These analytical methods have drawbacks, such as relative low selectivity, especially compared with the state-of-the-art analytical methods using mass spectrometry (MS) and nuclear magnetic resonance (NMR) spectroscopy [14].

MS is considered one of the cornerstone techniques for the analysis of both small and macro organic molecules [18]. Since its development in the 1980s, LC-MS has been utilized intensively in identifying ARPs and some recent studies are listed in Table 1 [19]. GC-MS is also reported for the detection of glycine-glucose-ARP [10,20]. However, GC-MS needs additional derivatization and is not suitable for massive detection [21].

MS/MS techniques or even MS^3^ techniques elevated the selectivity and structure identification based on every ion fragment of the molecular ion [22]. A recent study profoundly distinguished the standards of glycine-glucose- and proline-glucose-Schiff base, ARP and HRP by ESI-MS/MS [4]. Methods using stable isotope dilution assay with LC-MS/MS were also proposed [23,24]. Though the high cost of MS detection and onerous procedure bring certain impediments, the identification of small molecules like ARPs in a complex matrix becomes amenable and reliable, which is advantageous and has vast application.

The MS Library plays a big role in MS technique, for instance, the NIST library is useful in the analysis of essential oils. However, there is no specific library for Maillard reaction compounds. Researchers need to deduce the compound based on ion fragments and/or further confirm by NMR. Up to now, only a few attempts have been made to generate small libraries of sulfur-methyl thioesters but there is a lack of extensive use [25]. Thus, the development of a comprehensive library of Maillard reaction would be an important contribution.

**Table 1:** Recent application of LC-MS on ARP detection and improvement of ARP yield.

Initial System	Molecular Ratio	Method	ARP Yield (to Amino Acid/Peptide)	Detection	Ref.
Glycine-ribose, aqueous	1:1	CTR (pH 7.5, 100 °C, 60 min) + VD (80 °C, 20 min)	64.50%	HPLC-ELSD & NMR	[14]
Cysteine-xylose, aqueous	2:1	Heating (90 °C, 40 min) + spray drying (pH 9.5, inlet temperature 190 °C)	59.48%	HPLC-ELSD & NMR	[26]
Phenylalanine-xylose, aqueous	1:2	CTR (pH 7.4, 80 °C, 50 min) + VD (30 °C, 30 min)	47%	UPLC-MS & NMR	[27]
Carnosine-glucose, NADES	1:2	Glucose/sucrose/water = 1:1:9, NADES (80 °C, 2 h)	49%	LC-TOF-MS & NMR	[28]
Phenylalanine-xylose, aqueous	1:2	CTR (pH 7.4, 90 °C, 15 min) + VD (30 °C, 30 min)	47.23%	LC-TOF-MS & NMR	[29]
Glutathione-xylose, aqueous	1:2	VD (90 °C, 25 min)	67.98%	LC-TOF-MS & NMR	[30]
Glutamic acid-glucose, aqueous	1:8	Freeze drying + heating (pH 9, 90 °C, 80 min)	96.08%	UPLC-MS/MS & NMR	[17]
Carnosine-glucose, aqueous	1:13	Freeze drying + heating (pH 7, 90 °C, 80 min)	95%	UPLC-MS/MS & NMR	[17]
Glycine-glucose, aqueous	1:1	Freeze drying + heating (50 °C, 16 h)	>40%	LC & NMR	[31]
Alanine-xylose, aqueous	1:2	EGCG addition + VD (pH 7.5, 90 °C, 15 min)	95%	LC-TOF-MS & NMR	[32]
Phenylalanine-xylose, aqueous	1:2	CTR (pH 7, 90 °C, 40 min) + VD (80 °C, 100 min)	74.86%	LC-MS & NMR	[33]
Proline-glucose, aqueous	1:1	CTR (pH 7.4, 90 °C, 60 min, 4% Na_2_SO_3_) + VD (15 min)	69.15%	UPLC-MS/MS & NMR	[34]

CTR: controlled thermal reaction; VD: vacuum dehydration.

## 3. Occurrence in Foods

Starting from the 1950 s, ARPs have drawn attention, and an array of studies have focused on analyzing ARPs in foods since then. ARPs were first found in browned freeze-dried apricots using ion-exchange chromatography in 1958 [35]. Though the resulting spectrum is highly complicated, it proved the existence of ARPs. After that, purified glycine-, alanine- and valine-glucose-ARPs were present in beer malt and were also found in soy sauce together with isoleucine- and leucine-glucose-ARPs [36]. As shown in Table 2, seven amino acid-glucose ARPs were detected in cocoa, coffee, barley malt, wheat malt, wheat beer, bell pepper and tomato, of which tyrosine and histidine ARPs were found present for the first time in foods [24]. The drying of food accelerated ARP formation, and formed ARPs also degraded during roasting in coffee and cocoa. The highest ARP concentration is valine-glucose in unroasted cocoa and dried bell pepper at 342 and 3460 mg/kg, respectively. The results have been correlated with other studies and shown in Table 2 [37]. Eight ARPs existed in dried fruit and vegetables, milk powder, tomato juice and paste, and red peppers, and the total ranges from 1.36 to 3415 mg/100 g. The drying of tomato juice facilitated ARP formation, which was further increased under vacuum. Though amino acid-APRs are identified from foods, peptides like oligopeptides should also be a focus for their abundance in foods. Unfortunately, only a few studies paid attention and succeeded in detecting peptide-ARPs, such as carnosine-glucose-ARP from meat broth, which is mentioned in detail in Section 6. Therefore, more attention should be paid to oligopeptide-ARP analysis in future studies to fill the gap.

## 4. Traditional and Recent Advances in Preparation

Intensive papers have demonstrated various preparation methods since the discovery of ARPs. Traditional chemical preparation methods include two big categories using unprotected glucose or protected d-glucose. Protected glucose like 4,6-*O*-benzylidene glucose can readily form glycosylamine with the presence of a primary amine without acid catalyst and successive Amadori rearrangement. Others include peracetylated sugars and 2,3:4,5-di-*iso*-propylidene fructose, forming acetylated glycosylamine and 1-trifluoromethanesulfonyl-2,3:4,5-di-*iso*-propylidene fructose, respectively. These protected glucoses largely facilitate the synthesis of target compounds due to the prevention of side-product formation, whereas their reactivity differs from parent sugar and the cost would be dramatically elevated, and thus they are unsuitable for industrial application.

Three methods use intact glucose: the fusion method, syrup method, and reflux method, of which the last one is the most used synthetic pathway [38]. The refluxing method generally mixes amino acid and sugar in an organic solvent, such as methanol, which is then refluxed at low temperature for days, and the solvent is then eluted by a cation exchanger with an increasing concentration of trichloroacetic acid and gains a yield of around 20 to 30%. The fusion method was proposed by Amadori in 1931 by heating equimolar amine and sugar at dry state and 70–80 °C for 2 h [39]. The brown product then goes through crystallization by hot ethanol, obtaining a final yield around 10 to 30%. For the syrup method illustrated by Weygand in 1940 [40], a mixture of sugar, amine, acid catalyst: water (1:1.1–1.4:0.002–0.02:2.5–3) is heated up to 100 °C for 10 to 30 min and becomes a dark solution. Further purification by ion exchange resin and hot ethanol crystallization is applied. Until recent years, these methods were still applied for reliability and repeatability [12,41]. These traditional synthetic methods display relative low yield and involve cumbersome and demanding steps. Nonetheless, they still give reference values and build up the foundation for subsequent advanced preparation methods.

Based on the aforementioned classic preparation methods, it can be seen that the overall yield depends on two major steps for ARP preparation: ARP synthesis and purification. Roughly, the purification remains similar as yet. Normally, two-step purification consists of a preliminary round using an ion-exchange resin in the H^+^ form and a second-round using semipreparative reverse phase-HPLC [14].

The key to recent advances happens at the synthesis part. As shown in Table 1, a few studies use various methods to improve ARP yield successfully. The main point is to increase the formation of ARP and decrease the following degradation. Organic synthesis is gradually out of consideration due to health- and cost-considerations. Various research groups return to synthesis in water solution as shown in Table 1, whereas ARP synthesis yield in aqueous solution is normally quite low. The reason behind this is that higher water activity (a_w_) has a great impact on the kinetics of ARP formation, showing a negative correlation of ARP stability, especially in the range from 0.8 to 1 [31,42]. Kranz and Hofmann prepared a natural deep eutectic solvent (NADES) system with low water content and improved the yield of carnosine-glucose ARP yield to 49% after heating for 2 h at 80 °C [28]. Cui et al. proposed a combination of thermal controlled reaction and successive vacuum dehydration which could provide a synergistic effect and improved the yield of phenylalanine-xylose-ARP from 13.6% to 47.23% after 30 min of vacuum dehydration [29]. Such a yield improving method was also applied to various subsequent studies of the group, such as glycine-ribose-ARP (0.77% to 64.5%), glutathione-xylose-ARP (8.44% to 67.98%) and proline-glucose-ARP (3.63% to 69.15%) [14,30,34]. However, not all the system is suitable for controlled thermal dehydration coupled with vacuum dehydration. In the system of cysteine and xylose, the dominant product becomes 2-threityl-thiazolidine-4-carboxylic acid (TTCA) rather than ARP. The application of short-time spray dehydration is alternatively applied and shifted the equilibrium TTCA to ARP, increasing the ARP yield to 59.48% when the inlet temperature is 190 °C and aqueous pH is adjusted to 9.5 [26].

Besides vacuum dehydration and spray dehydration, freeze-drying has also been proved to increase ARP yield efficiently. Zhang et al. used a NADES system prepared by freeze lyophilization and thermal treatment for 80 min which further improved the yield of glutamic acid-glucose-ARP, carnosine-glucose-ARP to 96.08% and 95%, respectively [17]. Correlatedly, Devidek et al. compared the formation efficiency of glycine-glucose-ARP in solid state (dry mixed, freeze dried), aqueous state (solution (20% *w*/*w* in water) and slurry method (20–80% *w*/*w* in water)), and found that the freeze drying had the best ARP yield [31].

Moreover, the addition of polyphenol epigallocatechin gallate (EGCG) could further improve the ARP yield of alanine-xylose from 2 to 95%, by forming ARP-EGCG adducts and blocking subsequent degradation [32]. A contradictory point herein is that EGCG might affect later flavor generation though it can impressively increase the yield. Further research can be addressed to illustrate the actual impact of ARP-EGCG on flavor comparing with pure ARP.

Besides water activity, other important variables that affect the ARP yield include salt concentration, pH value, temperature and time, reactant ratio and concentration.

Salt addition slows down the reaction and thus has an unfavorable impact on ARP formation. Studies showed that the addition of 10% NaCl resulted in 30% browning inhibition and the presence of 13% NaCl decreased 28% of glucose involvement and led to an 18% reduction of ARP [42,43]. As for pH value, the traditional view illustrated that acid would catalyze the Amadori rearrangement formation in the fusion method and increase the final ARP yield [40,44]. Somewhat of a contradiction, Cui et al. indicated that the addition of sodium sulfite provided an optimized pH buffering effect (pH 7.4) and improved the yield of phenylalanine-xylose-ARP from 47.23 to 74.86% [33]. Basic condition favors the ARP formation of glycine-ribose and glycine-glucose [14,45]. These contrary results imply that pH value largely influences ARP yield and may vary in different amino acid-sugar systems. The optimal pH value should take into account for each specific ARP preparation. Similarly, temperature has a critical role. Maillard reaction is vigorous at a high temperature such as 160 °C, underlying that high temperature will rapidly push ARP to undergo subsequent reactions and should be avoided in ARP synthesis. Previous research utilized low temperatures such as 40 °C, but for a lengthy period: it may take up to 14 days to gain a sufficient amount of ARP [42]. Recent studies chose optimal temperatures like 80 and 90 °C and the reaction time was greatly shortened to a few hours combined with dehydration. Apart from these well-known factors, the ratio of amino group and sugar and concentration should also be contemplated. Theoretically, equimolar of amino group and reducing sugar can produce a corresponding molar of ARP, and a higher initial concentration can increase the overall production. However, intramolecular reactions will compete with the Maillard reaction and weaken the final yield [46]. The amino/sugar ratio can be 1:2 or 2:1 accordingly to protect amino or sugar for higher Maillard reaction involvement. A sugar overdose ratio such as 1:10 is also used in some studies, intending to ensure complete transformation of amino acid/peptides to ARPs, but also brings in interference from side products of sugar caramelization and degradation. As for the initial concentration, further supporting studies come from the formation of ARPs glutamic acid-glucose and proline-glucose [17]. Elevated initial amino acid concentration decreases the final yield to a certain level such as 11% or even 33%.

In general, recent advances in ARP preparation have optimized water activity by lowering the water content, adjusting pH value, balancing the thermal treatment temperature-time length, and avoiding salt addition. As for concrete application of the dehydration method, simultaneous vacuum dehydration and a thermal reaction is more economical and suitable for industrial-scale production. Nonetheless, one big drawback is that reactants tend to stick to the glass walls causing fluctuations of temperature and difficulties of removal. Meanwhile, freeze drying and spray drying need a lengthy equilibrium period to optimal a_w_ and represent high-cost input but are still applied in the pharmaceutical industry and food industry recently and should also be considered for their high ARP yield.

## 5. Stability

Stability is the most important factor affecting flavor quality, the decisive aspect of consumer acceptance. Flavor stability is affected by chemical reactivity itself, environmental factors such as light and oxygen, and influences from the food matrix, such as fat, metal ions, and radicals [47].

**Figure 1:**
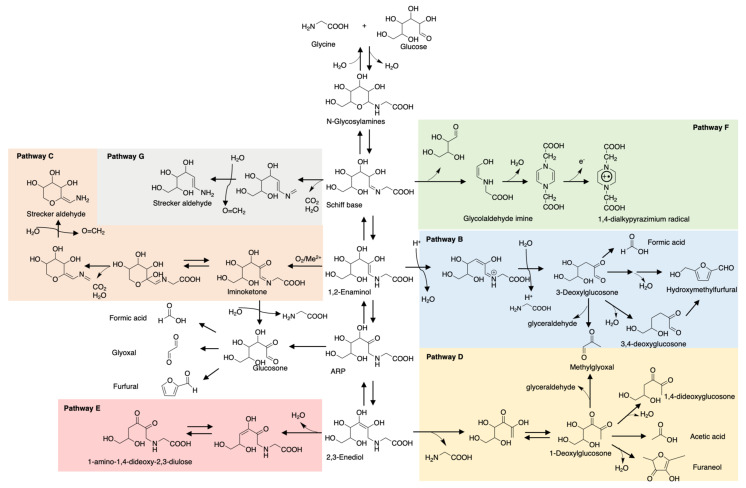
Degradation pathways of glycine-glucose-ARP. B. 1,2-Enolization pathway; C. Oxidative degradation pathway; D. 2,3-Enolization pathway; E. Elimination of C_4_-OH group of 2,3-enediol pathway; F. Cleavage of sugar moiety pathway; G. Decarboxylation pathway.

Maillard reaction products (MRPs) such as pyrrole and furan have been traditional key flavor compounds and food additives for decades [48]. However, one big defect of MRPs is their weak stability during storage and processing, especially the ones involved in thermal treatment. Pyranone, common in glucose- or lactose-rich food, and 3,4-dihydroxy-3-hexen-2,5-dione, a caramel odor, is significantly prone to degradation during short-term storage [49]. Furaneols, also known as strawberry furanone, are heat-labile and rapidly degrade preferably at low pH [50]. In contrast, studies have reported ARPs that were more stable during storage. Phenylalanine-xylose-ARP only reduced 6.49% after 2 months storage at room temperature while MRPs reduced more or less resulting in dramatic changes in flavor profile [27]. A consistent result is also reported for glutamic acid-glucose-ARP. In contrast to *N*-glycoside form rapidly degraded with acid catalyst, ^13^C NMR monitoring showed that glutamic acid-glucose-ARP in an aqueous solution maintained good stability for 3 days storage at pH 5 and room temperature, let alone with moderate or basic pH [51]. The opposite result for pH is shown in carnosine-glucose-ARP whose degradation rate increases with pH value increase [28]. This phenomenon depends on the amino acids/peptides’ chemical properties. Glutamic acid is acidic in aqueous solution while carnosine exists in basic form, thus are correspondingly more stable in acid and base conditions, respectively. Glycine, as the simplest amino acid, triggered more attention on its ARP investigation and model studies. As shown in Figure 1, the degradation pathways of glycine-glucose ARP in the aqueous system can be a reference for other ARP degradation studies [52].

Besides the abovementioned associations with intrinsic chemical nature and pH value, the stability of ARPs is also related to temperature, water activity, and the presence of a catalyst or stabilizer. Temperature plays a leading role in ARP stability. It shows a positive correlation with ARP degradation rate as more energy providence brings in higher reactivity and instability, so is the water activity [28]. The formation and decomposition kinetics of ARPs in freeze-dried carrots were studied and found that the lower the water activity, the higher Ea of formation while the higher water content, the higher Ea of decomposition. Hence, dilute solutions of ARPs are more sensitive to changes in temperature than concentrated solutions [53]. A supporting study showed that methionine-glucose-ARP had accelerated the degradation rate under high temperature and elevated formation of 3-DG, 1-DG, glucosone, and other dicarbonyls such as glyoxal and methylglyoxal [54]. Similarly, tryptophan-glucose-ARP showed a several times faster decomposition rate under high temperature and/or high water activity [55]. However, these studies mainly focused on high temperatures over 100 °C in order to imitate the flavor generation process, seldom investigation explores the stability of ARPs under storage conditions systematically.

Based on previous studies, adding ARPs as seasoning is a promising way to prolong shelf life for low water reactivity and storage under low or room temperature. However, extensive use of ARPs in different food matrices is an ultimate goal as good flavor additives. In addition to the neutral aqueous system, various food systems should be considered, for instance, the acidic fluid environment present in juice and soft drinks, base fluids such as sparkling spring water or meat broth, emulsion systems such as milk and meat broth, solid systems such as bread, cookies, and chips, and frozen instant products such as pizza and meat products. However, few studies reported the stability of ARPs in such models, which should be a priority before heat treatment and need further research in addition to intrinsic flavor generation ability.

## 6. Taste

**Figure 2:**
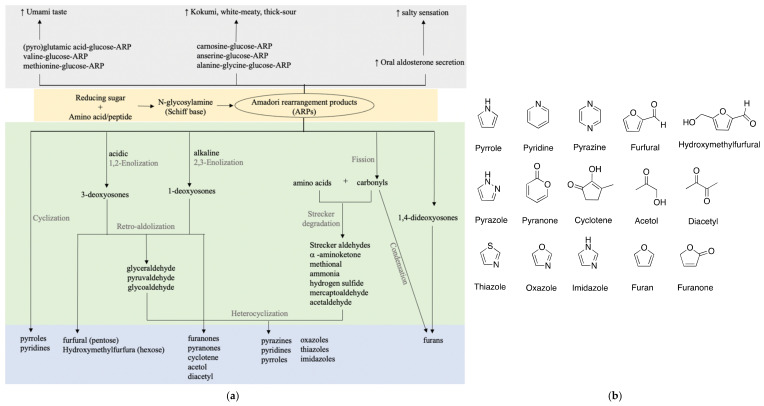
Taste and flavor formation of ARPs. (**a**) Taste enhancing properties and flavor formation of ARPs; (**b**) basic chemical structures of key flavors.

Many studies suggested that the ARPs of amino acids or peptides are correlated with umami or kokumi enhancing properties as shown in Figure 2. As glutamic acid and glucose are strongly correlated with umami taste, glutamic acid-glucose-ARP unsurprisingly showed a prominent umami-like taste at a low threshold of 1–2 mmol/L, close enough to that of monosodium glutamate (MSG) [51,56]. Accordingly, its aqueous solution exhibited distinct umami, seasoning, and bouillon-like taste similar to that containing equivalent MSG. Besides the purely chemical model, various studies have made an effort to isolate and identify umami-related compounds from food products, typically soy sauce. Only a few take a closer look at ARPs.

Pyroglutamic acid-, valine-, and methionine-glucose-ARPs were identified in a typical Japanese soy sauce named *koikuchi shouyu* at subthreshold concentrations, partially to contribute to the umami taste [57]. Similar umami enhancing was also reported for glutamic acid-glucose-ARP found in 25 commercial soy sauces [21]. A simultaneous quantification method of 20 ARPs in soy sauce using LC-MS was later proposed and verified in six types of soy sauce [58]. The omission of an ion-paring reagent and sample derivatization make it an applicable and propagable analytical method for ARP detection in broader food products. Besides soy sauce, umami or kokumi orosensation is always associated with meaty broths. Unsurprisingly, ARPs of dipeptide carnosine-glucose, anserine-glucose, alanine-glycine-glucose were elucidated to contribute to a white-meaty and thick-sour orosensation of chicken broth and traditional French meat-containing broth called Pot-au-Feu [59,60,61].

Roughly, ARPs exist in subthreshold concentrations individually in food products. Though not as the dominant and key taste/flavor component, they exhibit critical umami enhancing properties and the total sufficient amount and diversity affect the intensity and richness of the overall taste.

In addition to the umami taste, the salty taste is synchronously found in protein hydrolysates such as fish protein and peanut protein hydrolysates [62]. As mixtures of diverse amino acids and peptides, the protein hydrolysates may contain plenty of ARPs and correlate with a salty sensation. A supporting study was demonstrated in enzymatic hydrolysates of pea protein [63]. The concentration of Maillard reaction intermediates (MRIs) including ARPs derived from pea protein hydrolysates showed a positive correlation with umami and saltiness. Only 0.1% dosage of MRIs can attain 20% salt reduction. Further evidence was from direct evaluation of the pure ARPs. Proline-glucose-ARP is reported to provide a salty sensation as well as significant umami enhancement [34]. Electronic tongue and sensory evaluation confirmed that 0.4% of ARP addition contributed to 20% salt reduction in achieving equivalent saltiness. The mechanism comes from elevated oral aldosterone secretion stimulated by ARPs, which is strongly associated with salt sensitivity [64]. These studies illustrated that ARPs can also act as natural salt enhancers and address the call of an adult’s daily salt intake reduction to 5 g by World Health Organization.

Besides umami, salty and kokumi taste, ARPs may also have a potential association with other tastes such as sweet, bitter, and sour, because numbers of studies have illustrated correlations between these tastes and protein hydrolysates/peptides [65,66]. Further studies are needed to address these correlations in detail.

## 7. Flavor Generation

Flavor is of utmost importance in attracting consumers and maximizing food quality, hence flavor formation ability is a key parameter of ARP potential usage [47]. The generated flavor is highly linked with the amino acids/peptides of the ARPs. For instance, glycine-glucose-ARP provides cooked, roasted, and caramel-like flavor due to the large formation of pyrazines while glutamic acid-xylose-ARP gives biscuit-like and burnt aroma for the formation of low threshold aldehydes like nonanal and hexanal [66,67]. Phenylalanine-xylose-ARP facilitates the formation of flower-like flavor compounds during thermal treatment, contributing to a similar desirable flavor profile to a solution of MRPs [27,29]. ARPs exist in unfermented cocoa and increase significantly to mg/kg scale during short-term fermentation, and these ARPs are unequivocally precursors of cocoa odorants through the roasting process and generation of Strecker aldehydes like 3- or 2-methylbutanal, 3-(methylthio)propanal and phenylacetaldehyde [68]. Glycine and cysteine are two well-known amino acids in the preparation of sulfur-containing meaty flavors. However, glycine-glucose-ARP alone favors the formation of brown products rather than meaty flavor. The addition of cysteine merely facilitated the sulfur-containing compounds comparing to the cysteine-xylose-ARP/glycine system, resulting from the formation of relatively stable cyclic compounds such as 1,4-thiazane with ARPs and intermediates [69]. Adversely, cysteine-xylose-ARP alone can produce a low amount of meaty flavor compounds, whose formation was greatly facilitated by the addition of a small amount of glycine, leading to a subtotal amount more than twice the glycine-glucose-ARP/cysteine system. The most potent sulfur-containing compounds in these systems are 2-methylthiophene, 2-methyl-3-furanthiol, 3-mercapto-2-pentanone, 2-furfurylthiol, 3-thiophenethiol, and bis(2-methyl-3-furyl)-disulfide [69].

Besides of ARPs of amino acids, only few studies have probed into peptides. The glutathione-glucose system produced less roasted and nutty aroma with the same quantity as the glutamic acid-cysteine-glycine-glucose system, resulting from a slower release of hydrogen sulfide and ammonia participating in the subsequent volatile formation [70]. Glutathione-xylose-ARP was produced with a high yield of 67.98% by simultaneous dehydration reaction and produced more ketones, alcohols, and mainly furans (caramel-like and sweet flavor) than the mixture of the ARPs of the three amino acid constituents [30,71]. Additionally, the glutathione-xylose-ARP produced a few sulfur-containing compounds while the mixture of cysteine-xylose-ARP and three amino acid ARP mixture generated a potent meaty flavor, supporting the aforementioned suggestions that cysteine-xylose-ARP is capable of forming meaty flavors and the process is strengthened by glycine addition, possibly split from glycine-xylose-ARP.

Although they produced less pyrazines than the glycine-glucose system, diglycine-, triglycine- and tetraglycine-glucose produced several peptide-specific pyrazinones: 1,6-dimethy-2(1H)-pyrazinone, 1,5-dimethy-2(1H)-pyrazinone, 1,5,6-trimethy-2(1H)-pyrazinone [70]. Therefore, peptide-ARPs are likely to produce peptide-specific aromas and may reflect the reality better as fewer amino acids exist in food that are worthy of further study. Besides, peptides rather than amino acids dominate in real food systems and thus the investigation of peptide-ARPs may be a better reflection of reality. The sequence of the amino acids of peptides also impacts the flavor generation and needs more attention. Glycine-proline produced many more pyrrolizines and pyridine volatile compounds than proline-glycine at high temperature (180 °C), let alone the major production of glycine and proline mixture, including abundant proline-specific flavor 5-acetyl-2,3-dihydro-1H-pyrrolizine and 5-formyl-2,3-dihydro-1H-pyrrolizine [67].

## 8. Toxicology

Food safety is always a basic standard in the food industry and endures constant supervision by the government and the public. ARPs as promising flavor additives should be verified for their safety.

The Maillard reaction is associated with several thermal-induced toxicants, including heterocyclic amines (HCAs), acrylamide (AA), beta-carbolines, and advanced glycation end products (AGEs). Though existing at relatively low concentrations, these toxic compounds are considered either carcinogens or neurotoxins.

HCA formation depends on creatine, amino acids, and reducing sugars. HCAs exist in cooked proteinaceous foods, such as well-done and grilled meat. Exposure is also found in flavorings, beer, wine, and cigarette smoking [72]. HCAs are associated with tumor formation and cancers (breast, lung, stomach, and colon) in animal and human studies. [71] Formed by asparagine and reducing sugar, AA is linked with tumor formation and neurotoxic damage in animal studies [73]. Dietary consumption is mainly from Maillard-derived foods like fried potato, bakery, cereals, and coffee. Rapid absorption from the gastrointestinal tract after dietary exposure is followed by wide distribution to the tissues [74]. AA in the liver is metabolized to more reactive glycidamide towards DNA and proteins. Beta-carbolines norharman and harman, molecularly similar to Parkinson-inducing neurotoxin, are associated with Parkinson’s disease, tremor, and cancer [75,76]. Dietary and tobacco inhalation are the major sources of exogenous intake. The content of dietary intake is correlated with high-temperature processed food products [77]. Food products like fried, grilled, or flame-broiled meat generate large amounts of beta-carbolines [78,79]. Besides the drastic rapid Maillard reaction, a mild and long-term Maillard reaction is involved in products like soy sauce and vinegar also containing a relatively high content of beta-carbolines. Further evidence showed that they are specifically formed by the degradation of tryptophan-ARP but not from tryptophan [55].

Several amino acids such as phenylalanine, leucine, threonine, and serine are at a more important position in HCA formation [80], while that for AA and beta-carbolines are asparagine and tryptophan, respectively. Currently, the Food and Drug Administration (FDA) of the United States is still at the stage of the data-gathering process and has no regulation on HCAs, AA, and beta-carbolines. Nonetheless, since ARPs already finish the first step of the Maillard reaction, accelerated subsequent reactions are expected under high temperature and may lead to elevated production of these toxic substances. Whether these ARPs would facilitate toxicant formation remains unknown. Hence, extra caution should be drawn with the ARPs and their forming amino acids and further specific detailed mechanisms related to adverse effects of human health are required to clarify their relationship with food safety.

## 9. Conclusions

ARPs, owing to their advantages of good stability and efficiency in producing attractive fresh flavors, can act as potential food additives. The stability of ARPs is influenced by their chemical nature, pH value, temperature, water activity and food matrix. Modern analytical technologies such as LC/MS and NMR bring precise characterization of ARPs to reality. Advanced preparation methods such as synthesis, separation and drying processes can increase the yield of ARPs up to 95%, which would enable large-scale production in the future. The flavor formation is related to the amino acids/peptides of the ARPs. Peptide-ARPs can generate peptide-specific flavors, such as: 1,6-dimethy-2(1H)-pyrazinone, 1,5-dimethy-2(1H)-pyrazinone, and 1,5,6-trimethy-2(1H)-pyrazinone. It is worth noting that peptides are more abundant in food than amino acids but lack analysis on the corresponding ARPs. Besides flavor, ARPs are associated with umami and kokumi taste enhancing effects. However, the lack of systematic stability and toxicology studies need further research.

## Figures and Tables

**Table 2:** Concentration (mg/kg) of ARPs in different foods.

	**Fru-Ile**	**Fru-Tyr**	**Fru-Phe**	**Fru-His**	**Fru-Met**	**Fru-Leu**	**Fru-Val**	
Cocoa (unroasted)	104.00 ± 10.40	73.00 ± 2.92	104.00 ± 8.32	27.20 ± 1.36	2.33 ± 0.33	152.00 ± 12.16	342.00 ± 10.26	
Cocoa (roasted)	5.38 ± 0.32	3.04 ± 0.09	3.99 ± 0.28	0.62 ± 0.06	1.34 ± 0.12	6.47 ± 0.39	19.00 ± 0.95	
Coffee (green)	2.51 ± 0.13	0.18 ± 0.03	2.80 ± 0.81	0.36 ± 0.08	0.59 ± 0.08	4.65 ± 0.65	0.60 ± 0.03	
Coffee (roasted)	0.87 ± 0.14	ND	0.09 ± 0.02	15.33 ± 2.30	ND	0.81 ± 0.11	ND	
Barley malt	29.20 ± 2.04	11.00 ± 1.65	25.40 ± 3.05	13.10 ± 1.97	5.91 ± 0.95	33.80 ± 2.70	39.40 ± 4.73	
Wheat malt	27.80 ± 3.89	6.70 ± 0.34	21.10 ± 2.53	10.60 ± 1.06	4.31 ± 0.26	35.40 ± 5.00	148.00 ± 8.88	
Wheat beer	6.05 ± 0.12	2.91 ± 0.12	5.28 ± 0.05	2.77 ± 0.28	ND	11.50 ± 0.92	34.60 ± 0.00	
Bell pepper	0.10 ± 0.00	ND	0.08 ± 0.00	0.97 ± 0.20	0.10 ± 0.00	0.07 ± 0.01	0.58 ± 0.12	
Bell pepper (DW)	1.00	ND	0.80	9.70	1.00	0.70	ND	
Bell pepper powder	509.00 ± 15.27	210.00 ± 42.00	329.00 ± 26.32	405.00 ± 52.65	81.90 ± 4.10	592.00 ± 11.84	3460.00 ± 103.8	
Tomato	0.12 ± 0.01	ND	0.19 ± 0.03	0.57 ± 0.02	ND	0.16 ± 0.05	ND	
Tomato (DW)	2.40	ND	3.80	11.40	ND	3.20	ND	
Tomato powder	9.30 ± 1.67	7.96 ± 1.59	22.60 ± 3.39	45.00 ± 4.95	ND	9.60 ± 0.38	10.60 ± 1.70	
	**Fru-Arg**	**Fru-Ala**	**Fru-Phe**	**Fru-His**	**Fru-Met**	**Fru-Leu**	**Fru-Val**	**Fru-Glu**
Dried strawberries (DW)	8.4 ± 1.7 ^f^	30.5 ± 9.7 ^f^	9.0 ± 1.7 ^e^	12.5 ± 3.6 ^fg^	4.5 ± 2.2 ^e^	8.1 ± 1.6 ^e^	16.2 ± 4.5 ^d^	4.8 ± 2.0 ^d^
Dried bananas (DW)	2.3 ± 0.1 ^f^	1.6 ± 0.1 ^f^	0.2 ± 0.1 ^f^	8.2 ± 5.6 ^gh^	0.3 ± 0.2 ^e^	0.1 ± 0.1 ^e^	0.9 ± 0.3 ^d^	ND
Dried taro (DW)	2.3 ± 0.1 ^f^	74.5 ± 16.5 ^f^	11.4 ± 1.2 ^e^	16.6 ± 1.5 ^ef^	32.6 ± 8.1 ^c^	20.6 ± 8.9 ^e^	ND	ND
Milk powder (DW)	5.4 ± 0.3 ^f^	ND	0.2 ± 0.1 ^f^	0.2 ± 0.1 ^h^	0.7 ± 0.1 ^e^	0.2 ± 0.1 ^e^	ND	ND
Pulled figs (DW)	78.6 ± 3.7 ^e^	431.0 ± 43.8 ^d^	32.9 ± 2.7 ^d^	24.6 ± 1.4 ^e^	9.1 ± 0.7 ^de^	101.7 ± 24.1 ^d^	93.9 ± 20.3 ^c^	ND
Tomato juice (DW)	90.0 ± 0.8 ^e^	ND	10.0 ± 2.0 ^e^	10.0 ± 2.0 ^f^	16.0 ± 4.0 ^d^	10.0 ± 2.0 ^e^	ND	154.0 ± 1.5 ^b^
Tomato paste (DW)	156.0 ± 13.7 ^d^	295.2 ± 3.7 ^e^	428.7 ± 62.1 ^c^	186.0 ± 3.7 ^d^	46.3 ± 3.7 ^c^	262.5 ± 3.7 ^c^	103.3 ± 3.7 ^c^	1517.7 ± 3.7 ^a^
Red pepper I (DW)	2171.1 ± 103.5 ^b^	1291.5 ± 100.7 ^c^	974.3 ± 73.7 ^b^	454.4 ± 41.2 ^b^	300.0 ± 23.1 ^b^	1299.9 ± 90.8 ^b^	1341.5 ± 101.1 ^b^	ND
Red pepper II (DW)	1092.9 ± 101.1 ^c^	1952.2 ± 113.3 ^a^	836.1 ± 62.4 ^b^	264.5 ± 34.5 ^c^	240.2 ± 21.2 ^b^	1260.7 ± 123.1 ^b^	1453.9 ± 112.1 ^b^	ND
Red pepper III (DW)	24477.8 ± 212.6 ^a^	1479.7 ± 100.5 ^b^	2120.6 ± 183.7 ^a^	560.3 ± 49.2 ^a^	660.5 ± 65.2 ^a^	2613.6 ± 155.2 ^a^	2181.3 ± 143.5 ^a^	65.4 ± 8.7 ^c^

Data are mean values of triplicates (three separate work ups); Different letters (a, b, c, etc.) in the same column indicate significant (*p* < 0.05) differences between means. DW, dry weight; ND, not detected.

## Data Availability

Not applicable.
